# Phenolic Compounds from *Cinnamomum camphora* Roots: Extraction Optimization, Purification, Isolation, and Bioactivity Evaluation

**DOI:** 10.3390/molecules31091550

**Published:** 2026-05-06

**Authors:** Jinhua Long, Wei Zheng, Lu Liu, Yuting Pang, Yijia Zhang, Yuping Luan, Nan Xu

**Affiliations:** School of Pharmacy, Liaoning University of Traditional Chinese Medicine, Shenyang 110032, China; longjh_lzy@163.com (J.L.); zhengwei_lzy@126.com (W.Z.);

**Keywords:** *Cinnamomum camphora*, phenolics, extraction optimization, isolation, pancreatic lipase inhibition

## Abstract

Previous studies have demonstrated that *C. camphora* exhibits diverse bioactivities, but its roots, particularly their phenolic constituents, remain largely unexplored. In this study, we optimized the extraction and purification of total phenolics from *C. camphora* roots, isolated their chemical constituents, and evaluated their bioactivities. Response surface methodology yielded optimal conditions of 71% ethanol, 78 °C, and a liquid-to-solid ratio of 24:1 (mL/g), giving a total phenolic content of 3.60 mg/g. Purification with HPD-600 resin followed pseudo-second-order kinetics (*R*^2^ = 0.9987). From the enriched phenolic fraction, twelve phenolic compounds were isolated, seven of which are reported from *C. camphora* roots for the first time. The enriched fraction exhibited strong antioxidant activities (DPPH IC_50_ = 107.21 μg/mL; •OH IC_50_ = 130.7 μg/mL; O_2_^−^ IC_50_ = 141.70 μg/mL) and significant pancreatic lipase inhibitory activity (IC_50_ = 0.80 mg/mL). This integrated approach expands the chemical diversity of *C. camphora* roots and highlights their potential as a natural source of antioxidants and lipid-lowering agents.

## 1. Introduction

*Cinnamomum camphora* (L.) Presl, an evergreen tree of the Lauraceae family, occurs widely in tropical and subtropical regions of Asia, particularly in China, Japan, and Southeast Asia [1]. This species is renowned for its medicinal properties, with its roots, stems, leaves, and wood being extensively used in folk and traditional medicine to treat various ailments, including inflammatory conditions, pain, rheumatic arthritis, muscular strains, abdominal pain, cough, and respiratory disorders [2,3].

Previous pharmacological studies demonstrated that *C. camphora* exhibits diverse biological activities. The crude extracts and purified constituents showed significant anti-inflammatory effects through the inhibition of pro-inflammatory cytokines and the suppression of key signaling pathways [4,5,6,7]. They also displayed potent antioxidant activities, often surpassing those of commercial antioxidants [8,9,10]. Studies have also documented remarkable antimicrobial effects against various pathogens, including methicillin-resistant *Staphylococcus aureus* [11,12,13,14]. These diverse activities were often attributed to the rich array of secondary metabolites in *C. camphora*, with phenolic compounds playing a particularly important role [1,11,15]. Notably, phenolic compounds are well-recognized for their potential health benefits in preventing metabolic disorders such as hyperlipidemia, obesity, and cardiovascular diseases, making them a research hotspot in natural product chemistry [16,17,18]. Given the abundance of phenolic compounds in *C. camphora*, this species represents a promising candidate for the discovery of natural lipid-lowering agents.

Despite extensive studies on the aerial parts of *C. camphora*, the roots have received considerably less research attention, leaving their phenolic constituents largely unexplored, especially those with potential metabolic regulatory effects [19,20,21]. To fill this gap, the present study systematically investigated the phenolic constituents of *C. camphora* roots. Specifically, we optimized the extraction using response surface methodology, purified the total phenolics with HPD-600 macroporous resin (Shanghai yuanye Bio-Technology Co., Ltd., Shanghai, China), and isolated twelve compounds, seven of which are reported from *C. camphora* roots for the first time. Together, these findings expand the chemical diversity of this species and establish a scientific basis for its potential as a natural source of antioxidants and lipid-lowering agents.

## 2. Results

### 2.1. Results of One-Way Experiments on Total Phenolics Extraction

**Figure 1:**
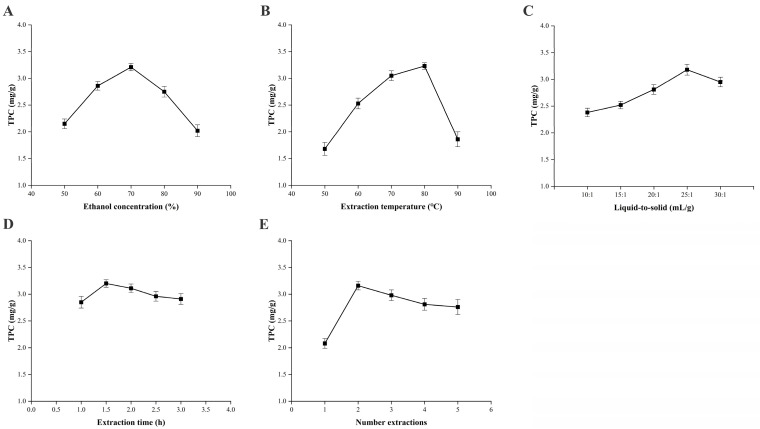
Effect of single factors on total phenolic content (TPC) extracted from *C. camphora* roots. (**A**) Ethanol concentration (50–90%, *v*/*v*); (**B**) extraction temperature (50–90 °C); (**C**) liquid-to-solid ratio (10:1 to 30:1, mL/g); (**D**) extraction time (1–3 h); (**E**) number of extraction cycles (1–5). Data were presented as mean ± SD (*n* = 3). Unless otherwise indicated, the extraction was performed under the optimal conditions determined from the single-factor experiments: temperature 80 °C, ethanol concentration 70%, liquid-to-solid ratio 25:1 (mL/g), extraction time 2 h, and two extraction cycles.

The effects of five factors on total phenolic content (TPC) were investigated. As illustrated in Figure 1, the optimal conditions were an ethanol concentration of 70%, an extraction temperature of 80 °C, a liquid-to-solid ratio of 25:1 (mL/g), an extraction time of 2 h, and two extraction cycles. Beyond these optimal parameters, further increases in each factor resulted in a decline in yield due to reduced polarity (ethanol), thermal degradation (temperature), impurity co-extraction (ratio), prolonged heating (time), or marginal improvement (cycles) [22]. These findings establish the foundation for subsequent response surface optimization.

### 2.2. Response Surface Optimization Results for Total Phenolic Extraction

A Box–Behnken design (BBD) with three independent variables—ethanol concentration (A), extraction temperature (B), and liquid-to-solid ratio (C)—and three levels was used to optimizes the extraction conditions for total phenolics from *C. camphora* roots. The experimental design and corresponding responses are presented in Table S2. Multiple regression analysis of the experimental data yielded the following second-order polynomial equation: *Y* = −31.8651 + 0.3375*A*^2^ + 0.0081*B*^2^ + 0.5927*C*^2^ + 0.0011*AB* − 0.0009*AC* + 0.0009*BC* − 0.0021*A*^2^ − 0.0034*B*^2^ − 0.0035*C*^2^, where *Y* represents the total phenolic content (mg/g), and *A*, *B*, and *C* denote the coded values of ethanol concentration (%), extraction temperature (°C), and liquid-to-solid ratio (mL/g), respectively. The analysis of variance (ANOVA) for the regression model is summarized in Table 1. The model demonstrates high significance, as evidenced by its extremely low *p*-value (<0.0001) and high *F*-value (150.45). The lack of fit is not significant (*p* = 0.1066 > 0.05), indicating that the model adequately fits the experimental data. The determination coefficient (*R*^2^ = 0.9949) and adjusted determination coefficient (*R*^2^_adj = 0.9882) suggest an excellent correlation between the predicted and actual values, with the model explaining 98.94% of the total variation [23]. The coefficient of variation (C.V. = 1.02%) and adequate precision (30.99) confirm the high reliability and accuracy of the model.

**Figure 2:**
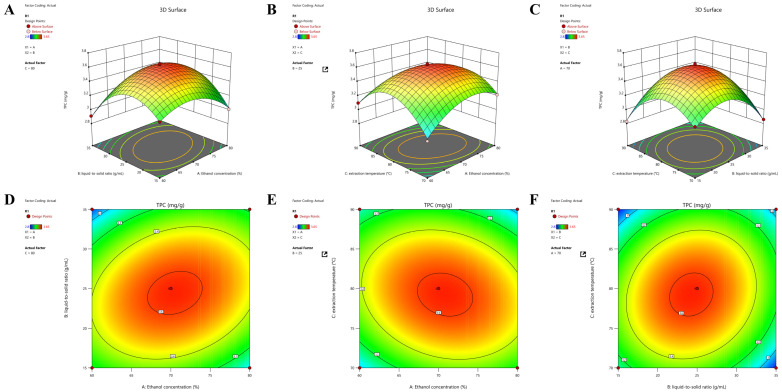
Three-dimensional response surface plots illustrating the interactive effects of extraction parameters on total phenols from *C. camphora* roots. (**A**,**D**) Effect of ethanol concentration (%) and extraction temperature (°C) at a fixed liquid-to-solid ratio of 25:1 (mL/g); (**B**,**E**) effect of ethanol concentration (%) and liquid-to-solid ratio (mL/g) at a fixed extraction temperature of 80 °C; (**C**,**F**) effect of extraction temperature (°C) and liquid-to-solid ratio (mL/g) at a fixed ethanol concentration of 70%. The color gradient from green to red indicates increasing total phenolic content (mg GAE/g dw).

Based on the response surface analysis (Figure 2), the optimal extraction conditions were predicted to be an ethanol concentration of 70.93%, an extraction temperature of 78.47 °C, and a liquid-to-solid ratio of 24.01:1 (mL/g). To enhance practical operability, these conditions were slightly modified to 71% ethanol, 78 °C, and 24:1 (mL/g). Verification experiments conducted under the modified conditions yielded an average total phenolic content of 3.60 ± 0.04 mg/g, which showed no significant difference from the predicted value of 3.62 mg/g, thereby confirming the model’s reliability. In addition, diagnostic plots, including the normal probability plot of residuals and the predicted vs. actual values plot (Figure S1), confirmed the adequacy of the regression model, with residuals normally distributed and predicted values in good agreement with experimental results.

### 2.3. Purification of Total Phenolics by HPD-600 Resin Column Chromatography

Based on a previous study on the purification of phenolics from *C. camphora* leaves [24], HPD-600 resin was selected for the enrichment of total phenolics from *C. camphora* roots.

#### 2.3.1. Adsorption Kinetics

**Figure 3:**
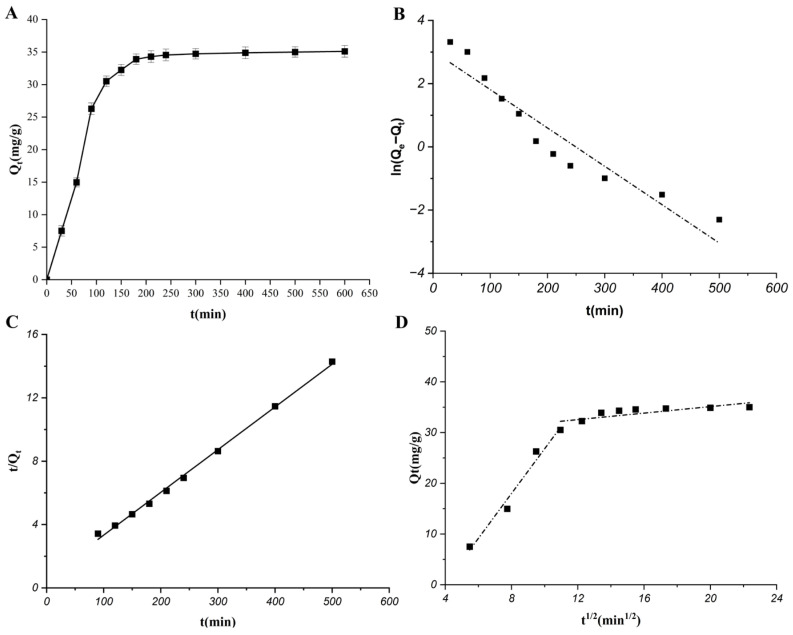
Adsorption kinetics of total phenolics from *C. camphora* roots on HPD-600 macroporous resin. (**A**) Experimental adsorption capacity over time (mean ± SD, *n* = 3, T = 25 °C, initial concentration 1.40 mg/mL). Linear correlations derived from the pseudo-first-order (**B**), pseudo-second-order (**C**), and intraparticle diffusion (**D**) kinetic models. The pseudo-second-order model shows the best fit (*R*^2^ = 0.9987).

The adsorption kinetics of total phenolics on HPD-600 resin were measured at 25 °C. As illustrated in Figure 3A, the adsorption rate increased rapidly during the initial 90 min, then gradually slowed and reached equilibrium after 150 min, with an equilibrium adsorption capacity of 34.33 mg/g. Three kinetic models were fitted to the experimental data (Figure 3B–D). The pseudo-second-order model provided the best fit (*R*^2^ = 0.9987), with a calculated equilibrium adsorption capacity of 34.72 mg/g, which aligns closely with the experimental value of 34.33 mg/g. The pseudo-first-order model also demonstrated good correlation (*R*^2^ = 0.9853). The intraparticle diffusion model exhibited multilinearity, characterized by two distinct stages (*R*^2^_1_ = 0.9613, *R*^2^_2_ = 0.9921). The plots did not pass through the origin (*C*_1_ = −8.15, *C*_2_ = 23.91), indicating that the rate-limiting step involved both boundary layer diffusion and intraparticle diffusion.

These results suggest that the adsorption of total phenolics onto HPD-600 resin followed a pseudo-second-order kinetic model, indicating that the adsorption process was dominated by chemisorption.

#### 2.3.2. Leakage Curves

**Figure 4:**
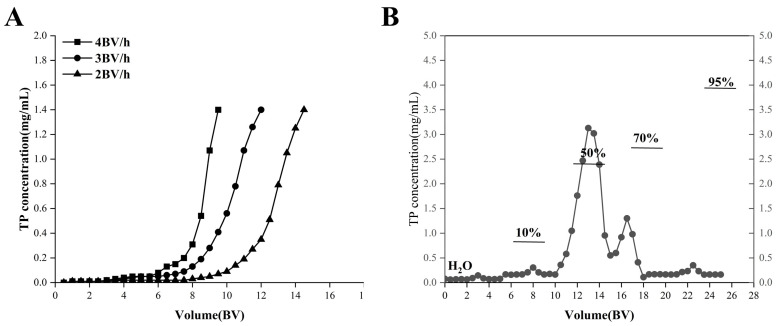
Breakthrough and elution profiles of total phenolics on HPD-600 resin. (**A**) Leakage curves at different flow rates (2, 3, and 4 BV/h). The leakage point (10% of feed concentration) was reached at 9.5 BV (2 BV/h), 8 BV (3 BV/h), and 6.5 BV (4 BV/h). (**B**) Gradient elution profile: 5 BV each of water, 10% ethanol, 50% ethanol, 70% ethanol, and 95% ethanol at a flow rate of 3 BV/h. The fractions eluted with 50% and 70% ethanol were collected as the enriched phenolic extract. BV: bed volume (20 mm × 200 mm column).

Figure 4A illustrates that leakage occurred more rapidly at higher feed speeds, as lower feed speeds provided increased contact time with the resin. The leakage points at feed speeds of 2, 3, and 4 BV/h are 9.5, 8, and 6.5 BV, respectively. Consequently, 2 BV/h was identified as the optimal feed speed, corresponding to a feed volume of 9.5 BV.

#### 2.3.3. Elution Curves

Phenolics were desorbed from the HPD-600 resin column using a gradient elution procedure. As shown in Figure 4B, water (5 BV) and 10% ethanol (5 BV) initially removed polar contaminants. Subsequently, 50% ethanol (5 BV) eluted the phenolic-enriched fraction, while 70% ethanol (5 BV) eluted only a small amount of phenolics. The fractions obtained from the 50% and 70% ethanol were collected, concentrated, and freeze-dried to yield the enriched phenolic extract. The purified extract obtained under the optimal conditions contained total phenolics at 52.3 mg GAE/g and total flavonoids at 22.6 mg QE/g.

### 2.4. Isolation and Structural Elucidation of Phenolic Compounds from C. camphora Roots

#### 2.4.1. UPLC-ESI-QTOF-MS/MS Profiling of the Total Phenolic Extract

The total phenolic extract was analyzed by UPLC-ESI-QTOF-MS/MS. Based on accurate mass measurements and MS/MS fragmentation patterns, 45 compounds were tentatively identified, including phenolic acids and flavonoids (Table S1). The total ion chromatograms are shown in Figure S2. These results indicate that phenolic acids and flavonoids represent the dominant constituents in the extract, thereby guiding subsequent targeted isolation.

#### 2.4.2. Isolation and Structural Elucidation

**Figure 5:**
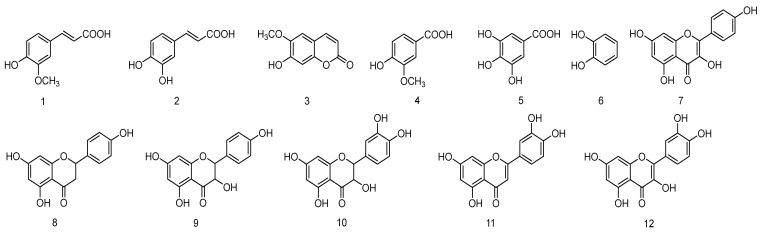
Chemical structures of compounds **1**–**12** isolated from the phenolic extract of *C. camphora* roots.

Guided by LC-MS/MS profiling, the enriched phenolic fraction was purified by repeated column chromatography, resulting in the isolation of twelve compounds (Figure 5): ferulic acid (**1**), caffeic acid (**2**), scopoletin (**3**), vanillic acid (**4**), gallic acid (**5**), catechol (**6**), kaempferol (**7**), naringenin (**8**), dihydrokaempferol (**9**), taxifolin (**10**), luteolin (**11**), and quercetin (**12**). Among these, seven compounds (**1**, **2**, **4**, **5**, **8**, **9**, **10**) were reported from *C. camphora* roots for the first time. Due to compound-dependent ionization efficiencies, peak intensity in ESI-MS does not quantitatively reflect actual compound abundance. Therefore, the 12 compounds reported herein represent the major phenolic constituents of the extract in terms of isolated mass. The remaining 33 tentatively identified compounds (Table S1) were present in trace amounts and could not be isolated due to material limitations.

Moreover, the structures of compounds **1**–**12** were confirmed by comparison of their HR-ESI-MS and NMR data with literature values. For example, ferulic acid (**1**) exhibited a characteristic trans-double bond (*δ*_H_ 7.59, d, *J* = 15.9 Hz, H-7; *δ*_H_ 6.30, d, *J* = 15.9 Hz, H-8) and a methoxy group (*δ*_H_ 3.91, s, 3-OCH_3_). Caffeic acid (**2**) showed analogous trans-double bond signals (*δ*_H_ 7.53, d, *J* = 15.9 Hz, H-7; *δ*_H_ 6.22, d, *J* = 15.9 Hz, H-8). The flavone skeleton of quercetin (**12**) was confirmed by the ABX system of ring B (*δ*_H_ 7.67, d, *J* = 2.2 Hz, H-2′; 7.54, dd, *J* = 2.2, 8.5 Hz, H-6′; 6.88, d, *J* = 8.5 Hz, H-5′) and the typical H-6/H-8 signals (*δ*_H_ 6.18, d, *J* = 2.0 Hz, H-6; 6.40, d, *J* = 2.0 Hz, H-8). The NMR data for the remaining compounds are provided in the Supplementary Materials.

### 2.5. Antioxidant and Pancreatic Lipase Inhibitory Activities

**Figure 6:**
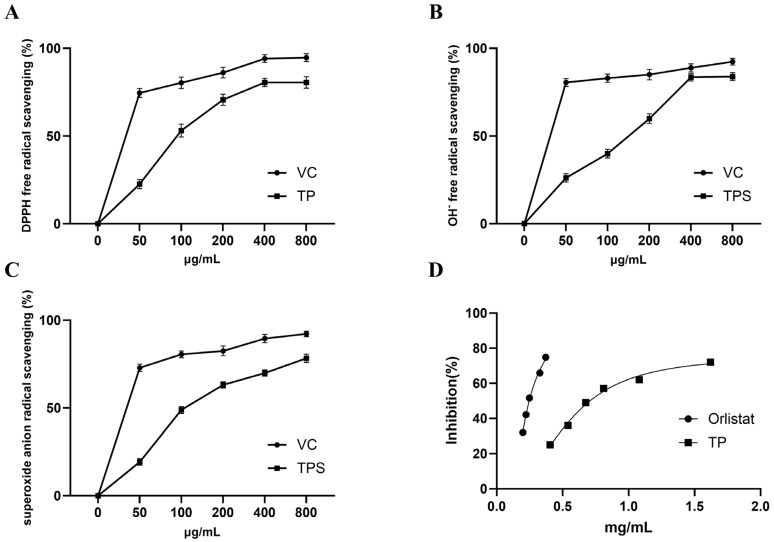
In vitro antioxidant and pancreatic lipase inhibitory activities of the enriched phenolic extract from *C. camphora* roots. (**A**) DPPH radical scavenging activity (IC_50_ = 107.21 ± 2.03 μg/mL); (**B**) hydroxyl radical (•OH) scavenging activity (IC_50_ = 130.70 ± 2.12 μg/mL); (**C**) superoxide anion (O_2_^−^) radical scavenging activity (IC_50_ = 141.70 ± 2.15 μg/mL). Ascorbic acid was used as the positive control. (**D**) Pancreatic lipase inhibitory activity (IC_50_ = 0.80 ± 0.05 mg/mL); orlistat was used as the positive control (IC_50_ = 0.22 mg/mL). Data are presented as mean ± SD (*n* = 3).

The antioxidant activities of total phenolics were evaluated using DPPH, superoxide anion, and hydroxyl radical scavenging assays. The half-maximal inhibitory concentration (IC_50_) values were 107.21 ± 2.03, 141.70 ± 2.15, and 130.70 ± 2.12 μg/mL, respectively. Total phenolics also exhibited significant pancreatic lipase inhibitory activity in a dose-dependent manner (Figure 6), with an IC_50_ of 0.80 ± 0.05 mg/mL, while the positive control orlistat showed an IC_50_ of 0.22 mg/mL under the same experimental conditions.

## 3. Discussion

In this study, we systematically investigated the phenolic constituents of *C. camphora* roots, a plant part that has received considerably less research attention than its aerial parts. For the first time, we optimized the extraction and purification of total phenolics from this tissue using response surface methodology and macroporous resin chromatography, isolated twelve phenolic compounds (seven of which are new to this species), and evaluated their antioxidant and pancreatic lipase inhibitory activities. These findings significantly expand the chemical profile of *C. camphora* roots and suggest their potential for further evaluation as natural antioxidants and lipid-lowering agents.

The adsorption of total phenolics onto HPD-600 resin followed a pseudo-second-order kinetic model (*R*^2^ = 0.9987), indicating chemisorption as the rate-limiting step. This result aligns with previous findings on the purification of phenolics from *C. camphora* leaves [25] and other medicinal plants [24], suggesting that HPD-600 resin is broadly suitable for phenolic enrichment from *Cinnamomum* species. The optimal loading conditions (2 BV/h, 9.5 BV) and elution with 50% and 70% ethanol established in this study provide practical parameters for large-scale preparation. As shown in Section 2.3, the resin achieved an equilibrium adsorption capacity of 34.33 mg/g for total phenolics, and gradient elution with 50% and 70% ethanol yielded efficient desorption. The successful isolation of diverse phenolic compounds from these eluates, including simple phenolic acids (e.g., gallic acid, vanillic acid) and flavonoids (e.g., quercetin, luteolin), further confirms that HPD-600 resin captures a broad range of phenolic structures. Together, these results demonstrate that HPD-600 combines good adsorption, efficient desorption, and broad compound coverage, making it well-suited for this application.

The optimal extraction conditions (71% ethanol, 78 °C, liquid-to-solid ratio 24:1 mL/g) yielded a total phenolic content of 3.60 mg GAE/g dw. Compared with previous studies on *C. camphora* leaves (TPC of 2.45–4.12 mg GAE/g dw under similar conditions [16,17]), this value indicates that roots are a viable alternative source of phenolics. Furthermore, it falls within the range reported for other Lauraceae plants, such as *Cinnamomum cassia* bark (2.89–4.53 mg GAE/g dw) [26]. While this value is lower than that of highly optimized commercial sources like green tea (150–300 mg GAE/g dw) [22], it is comparable to several medicinal plants already used in nutraceutical formulations, such as Plantago depressa (0.45 mg GAE/g dw) [23]. Therefore, while the absolute content is moderate, the significant bioactivities observed (as detailed above: DPPH IC_50_ = 107.21 μg/mL; pancreatic lipase IC_50_ = 0.80 mg/mL) suggest that the extract has promising potential for nutraceutical applications, particularly as a lipid-lowering functional ingredient.

The isolated phenolic compounds included coumarins (scopoletin) and flavonoids (kaempferol, naringenin, quercetin), which align with the characteristic secondary metabolite profiles of the Lauraceae family [20,26]. Notably, the coexistence of C6–C1 (gallic acid, vanillic acid), C6–C3 (ferulic acid, caffeic acid), and C6–C3–C6 (flavonoids) skeletons in the same tissue reflects the diverse biosynthetic capabilities of *C. camphora* roots. These findings enrich the chemical library of this species and provide valuable chemotaxonomic markers for distinguishing *C. camphora* from other *Cinnamomum* species.

The total phenolic fraction exhibited potent radical scavenging activity, with IC_50_ values of 107.2 μg/mL (DPPH), 130.7 μg/mL (•OH), and 141.7 μg/mL (O_2_^−^). These values are comparable to, or in some cases better than, those reported for other medicinal plants. For example, the DPPH IC_50_ of *C. camphora* leaf extract was reported as 185.30 μg/mL [17], indicating that roots may be a superior source of natural antioxidants. The antioxidant activity of phenolic compounds is determined by both the number and position of hydroxyl groups. Among the 12 compounds isolated in this study, three distinct structural types can be distinguished: (1) ortho-dihydroxy (catechol) structures on the B-ring (quercetin, luteolin, and caffeic acid); (2) trihydroxy phenolic structures (gallic acid); and (3) monohydroxy or methoxylated structures (vanillic acid, ferulic acid, and catechol). Their expected antioxidant potency follows the order type (1) ≈ type (2) ≥ type (3), because ortho-dihydroxy and trihydroxy motifs enable efficient electron delocalization and hydrogen donation, allowing for the formation of stable quinone radicals [27,28,29]. In contrast, compounds lacking these features (e.g., vanillic acid) are expected to be much weaker radical scavengers. The potent activity of the total phenolic fraction (DPPH IC_50_ = 107.2 μg/mL) is fully consistent with this interpretation, as this fraction is rich in compounds of types (1) and (2).

The total phenolic fraction inhibited pancreatic lipase with an IC_50_ of 0.80 mg/mL, compared to 0.22 mg/mL for orlistat, a clinically used anti-obesity drug [30], indicating that the extract is moderately active. To better contextualize this activity, we compared it with other well-characterized plant-derived pancreatic lipase inhibitors. The IC_50_ value of *C. camphora* root extract (0.80 mg/mL) is comparable to that of lotus (Nelumbo nucifera) leaf total flavonoids (0.72 mg/mL) [18], but lower than that of ginkgo (Ginkgo biloba) leaf total flavonoids (0.45 mg/mL) [19]. These differences may be attributed to variations in the composition and structural features of flavonoids among different plant species [31]. Notably, the activity of *C. camphora* root extract also falls within the range reported for Camellia sinensis (IC_50_ = 0.52–1.15 mg/mL) [32]. The observed pancreatic lipase inhibitory activity can also be rationalized by structural features. Flavonoids with hydroxyl groups at positions 5 and 7 on the A-ring and ortho-dihydroxy structures on the B-ring (e.g., quercetin, kaempferol) are known to interact with the catalytic site of pancreatic lipase via hydrogen bonding and hydrophobic interactions [33]. In contrast, flavonoids lacking the B-ring catechol structure (e.g., naringenin) are expected to be weaker inhibitors. Phenolic acids such as caffeic acid and ferulic acid may also contribute, but their activity is generally lower than that of ortho-dihydroxy flavonoids. The moderate activity of the total phenolic fraction (IC_50_ = 0.80 mg/mL) reflects the combined effects of these structural classes.

Nevertheless, several limitations should be acknowledged. First, the extraction optimization relied solely on total phenolic content (TPC) as the response variable, without parallel measurement of total flavonoid content (TFC). Future studies may consider using both TPC and TFC as complementary parameters for a more comprehensive evaluation. Second, all bioactivity evaluations were conducted in vitro using cell-free systems. Thus, the current data are preliminary, and further validation using cellular and animal models is required to confirm the lipid-lowering effects and safety profile. Third, although LC-MS/MS profiling detected 45 compounds, only 12 were isolated and characterized. These 12 compounds were prioritized based on their actual material availability during column chromatography; the remaining 33 compounds were present in trace amounts and could not be isolated due to material limitations. Nevertheless, many of these unisolated compounds belong to classes known for strong bioactivity, including flavonoids (e.g., myricetin, eriodictyol), curcuminoids, and phenolic acids. Their presence, albeit in trace amounts, may contribute to the observed antioxidant and lipase inhibitory activities through additive or synergistic effects with the major isolated compounds. Additionally, due to the limited yield of individual isolates (ranging from 5.1 mg to 12.5 mg), their individual bioactivities were not evaluated in this study. Future work will focus on isolating these minor components using larger sample quantities, evaluating their individual bioactivities, and establishing structure–activity relationships to identify the most potent antioxidant and pancreatic lipase inhibitors.

## 4. Materials and Methods

### 4.1. Materials and Chemicals

The *Cinnamomum camphora* roots (batch number: 250312) were purchased from Hainan Huluwa Pharmaceutical Group Co., Ltd. (Haikou, China). The plant material was further authenticated by Professor Liang Xu, School of Pharmacy, Liaoning University of Traditional Chinese Medicine, based on morphological characteristics according to the Flora of China. A voucher specimen (No. 20250401) has been deposited at the Herbarium of Liaoning University of Traditional Chinese Medicine.

### 4.2. Determination of Total Phenols and Total Flavonoids

The chemicals and reagents used in this study (Folin–Ciocalteu, Na_2_CO_3_, NaNO_2_, gallic acid, AlCl_3_, NaOH, and quercetin) were purchased from Shanghai Aladdin Biochemical Technology Co., Ltd. (Shanghai, China).Total phenolic content was determined by employing the Folin–Ciocalteu method [29]. Briefly, 2.0 mL of the sample solution was mixed with 0.5 mL of Folin–Ciocalteu reagent and allowed to stand for 5 min. Then, 1.5 mL of 10% Na_2_CO_3_ solution was added, and the mixture was incubated at 25 °C for 20 min. The absorbance was measured at 760 nm. A calibration curve was constructed using gallic acid standards (50–250 μg/mL), yielding the equation: *y* = 2.8144*x* + 0.0106 (*R*^2^ = 0.9991). Results were expressed as milligrams of gallic acid equivalents per gram of dry weight (mg GAE/g dw).

Total flavonoid content was determined using a modified version of the method described by Chang et al. [30]. Briefly, 1 mL of the sample solution was mixed with 1.4 mL of distilled water and 300 μL of 5% NaNO_2_ solution. After 5 min of incubation, 300 μL of 10% AlCl_3_ solution was added, followed by 2 mL of 1 M NaOH and 2.8 mL of distilled water. The absorbance was measured at 415 nm. A calibration curve was constructed using quercetin standards (1.042–33.344 μg/mL), yielding the equation: *y* = 0.0823*x* + 0.0117 (*R*^2^ = 0.9985). Results were expressed as milligrams of quercetin equivalents per gram of dry weight (mg QE/g dw).

All measurements for both total phenolic and total flavonoid content were performed in triplicate, and data are presented as mean ± SD.

### 4.3. One-Way Experimental Design for Total Phenolics Extraction

The study investigated the effects of various factors on phenolic yield, including ethanol concentration (50%, 60%, 70%, 80%, and 90%), extraction temperature (50 °C, 60 °C, 70 °C, 80 °C, and 90 °C), extraction time (1, 1.5, 2, 2.5, and 3 h), liquid-to-solid ratio (10:1, 15:1, 20:1, 25:1, and 30:1 mL/g), and the number of extractions (1, 2, 3, 4, and 5).

### 4.4. Response Surface Methodology (RSM) Experiments

Based on the results of the single-factor experiments, the optimization was conducted with the TPC in the extract set as the response value (*Y*), and the ethanol concentration (A), extraction temperature (B), and liquid-to-solid ratio (C) serving as the independent variables, respectively. The design of factors and levels for the response surface test is shown in Table 2.

### 4.5. Purification of Phenolics Using Macroporous Resins

#### 4.5.1. Kinetic Curves of Adsorption and Desorption

HPD-600 macroporous resin (1.0 g) was weighed, and 20 mL of the *C. camphora* roots extract was added. The mixture underwent shaking in a constant temperature oscillation water bath at 100 rpm and 25 °C. The total phenolic concentration was analyzed at predetermined adsorption times (0, 30, 60, 90, 120, 150, 180, 240, 300, 400, 500, and 600 min) until equilibrium was achieved. To investigate the adsorption mechanism and provide a theoretical foundation for the rational design of an optimal adsorption system, three prevalent kinetic models were applied:

Pseudo-first-order model:

ln (*Q*_e_ − *Q*_t_) = ln *Q*_e_ − k_1_*t* (1)

Pseudo-second-order model:

t/*Q*_t_ = t/*Q*_e_ + 1/*k*_2_*Q*_e_^2^ (2)

Intraparticle diffusion model:

*Q*_t_ = *k_i_t*^1/2^ + *C*_i_ (3)

where *Q*_t_ (mg/g) is the adsorption capacity at time *t*, *k*_1_ (min^−1^) and *k*_2_ [g/(mg⋅min)] are the rate constants, *k*_i_ [mg/(g⋅min^1/2^)] is an intraparticle diffusion rate constant; and *C*_i_ (mg/g) is the boundary layer thickness constant [27].

#### 4.5.2. Leakage and Elution Curves

A HPD-600 macroporous adsorption resin column (*ϕ* 20 mm, diameter-to-height ratio 1:10) served for purification optimization. For the leakage curves, the phenolic extract (1.40 mg/mL) was loaded at flow rates of 2, 3, and 4 BV/h. The effluent was collected in 0.5 BV fractions, and the total phenolic content was measured until breakthrough occurred. The leakage point was defined as the volume at which the phenolic concentration in the effluent reached 10% of that in the feed solution. For the elution curves, after loading under optimal conditions, the column underwent sequential elution with 5 BV of water, 5 BV of 10% ethanol, 5 BV of 50% ethanol, 5 BV of 70% ethanol, and 5 BV of 95% ethanol at a rate of 3 BV/h. The fractions containing 50% and 70% ethanol were combined, concentrated, and freeze-dried to yield the enriched phenolic extract.

### 4.6. UPLC-MS/MS Analysis

The total phenolic extract obtained under optimal conditions underwent analysis using an Agilent 1290 Infinity II UHPLC system coupled with an Agilent 6545 Q-TOF mass spectrometer (Agilent Technologies, Santa Clara, CA, USA). Chromatographic separation occurred on an Agilent Poroshell 120 EC-C18 column (4.6 mm × 100 mm, 2.7 μm) maintained at 35 °C. The mobile phase comprised acetonitrile (A) and 0.1% formic acid in water (B), employing a gradient elution program as follows: 0–10 min, 5–50% A; 10–25 min, 50–70% A; 25–30 min, 70–100% A. The flow rate was set at 0.4 mL/min, and the injection volume was 5 μL. Mass spectrometry utilized a dual Agilent Jet Stream electrospray ionization (AJS ESI) source operating in both positive and negative ion modes. The mass range was established from *m*/*z* 50 to 1000. The operating parameters were as follows: gas temperature, 320 °C; drying gas flow rate, 8 L/min; nebulizer pressure, 35 psig; sheath gas temperature, 350 °C; sheath gas flow rate, 11 L/min; capillary voltage, 3500 V; nozzle voltage, 1000 V; fragmentor voltage, 180 V. Data acquisition and processing were performed using Agilent Mass Hunter Workstation software (Version 10.0).

### 4.7. Isolation and Identification of Chemical Constituents

The enriched phenolic fraction (26.5 g) was subjected to silica gel column chromatography (CC) and eluted with a gradient of petroleum ether–ethyl acetate (100:0 → 0:100, *v*/*v*) to yield eight fractions (Fr. A-H).

Compound **1** (12.5 mg) was crystallized from Fr. B (3.1 g). Fr. C (2.8 g) was fractionated by preparative HPLC (MeOH–H_2_O = 85:15, 1.5 mL/min) to yield compounds **2** (9.8 mg) and **3** (5.7 mg). Fr. D (3.2 g) was subjected to repeated silica gel CC (PE–EtOAc, 80:20 → 0:100) and Sephadex LH-20 CC to afford compounds **4** (6.7 mg), **5** (7.5 mg), and **6** (5.5 mg). Compound **7** (5.4 mg) and **8** (5.1 mg) were isolated from Fr. F (3.8 g) by preparative HPLC (MeOH–H_2_O = 80:20, 1.5 mL/min). Compounds **9** (11.2 mg) and **10** (7.4 mg) were obtained from Fr. G (5.4 g) by Sephadex LH-20 CC and preparative HPLC (MeOH–H_2_O = 77:23, 1.5 mL/min). Fr. H (5.6 g) was fractionated by Sephadex LH-20 CC (GE Healthcare, Marlborough, MA, USA) to yield four subfractions (Fr. H1–H4). Compounds **11** (5.1 mg) and **12** (6.4 mg) were purified from Fr. H2 by preparative HPLC (MeOH–H_2_O = 75:25, 1.5 mL/min). All isolated compounds were identified by HR-ESI-MS and 1D NMR analyses (See Supplementary Materials).

### 4.8. Analysis of Anti-Oxidation Activity

The antioxidant activity was evaluated using DPPH, hydroxyl (•OH), and superoxide anion (O_2_^−^) radical scavenging assays, with ascorbic acid as the positive control [33]. For all assays, the sample solution and standard were tested at various concentrations, and a blank control was prepared by replacing the sample with an equal volume of distilled water to correct for background absorbance.

For the DPPH assay, 100 μL of DPPH was mixed with 100 μL of the sample solution or standard. The reaction mixture was incubated in dark for 5 min, and the optical density was measured at 517 nm.

For the •OH assay, the reaction mixture contained 100 μL each of the sample solution or standard (various concentrations), 6 mM FeSO_4_, 6 mM H_2_O_2_, and 6 mM salicylic acid. After incubation at 37 °C for 30 min, the absorbance was measured at 510 nm.

For the O_2_^−^ assay, the reaction mixture contained 100 μL each of the sample solution (various concentrations), 0.1 M phosphate buffer (pH 7.4), 0.1 mM NBT, 0.1 mM NADH, and 0.03 mM PMS. After incubation at 25 °C for 5 min, the absorbance was measured at 560 nm.

The scavenging rate was calculated as: Scavenging (%) = [(A_0_ − A_1_)/A_0_] × 100%, where A_0_ and A_1_ are the absorbances of the control and sample, respectively. The half-maximal inhibitory concentration (IC_50_) value was calculated by nonlinear regression using GraphPad Prism [32].

### 4.9. Pancreatic Lipase Inhibitory Activity Assay

Pancreatic lipase inhibitory activity was determined according to a previously reported method with slight modifications [33]. Briefly, 10 μL of total phenolics solution (0.4–1.6 mg/mL), 80 μL of pancreatic lipase (6.5 mg/mL), and Tris-HCl buffer (pH 8.0) were mixed in a 96-well plate to a final volume of 100 μL. After incubation at 37 °C for 20 min, the reaction was initiated by adding 10 μL of p-NPP (3 mg/mL) and further incubated at 37 °C for 20 min. The control group was prepared by replacing the enzyme solution with buffer. Absorbance was measured at 405 nm using a microplate reader. All tests were performed in triplicate.

The inhibition rate was calculated as follows:

$$\mathrm{Inhibition}\left(\%\right)\;=\;[1\;-\;\frac{A\;-\;a}{B\;-\;b}]\;\times \;100\%$$

where A and a represent the absorbance of the control and negative control without inhibitor, respectively, and B and b represent the absorbance of the sample and negative control with inhibitor, respectively. All experiments were performed in triplicate, and results were expressed as mean ± standard deviation (SD).

### 4.10. Statistical Analysis

The response surface methodology (RSM) results were analyzed using Design-Expert software (version 13.0, Stat-Ease Inc., Minneapolis, MN, USA), with model significance determined by ANOVA (Table 1). IC_50_ values for antioxidant and pancreatic lipase inhibitory activities were calculated by nonlinear regression using GraphPad Prism 9.0 (GraphPad Software, San Diego, CA, USA).

## 5. Conclusions

In this study, the extraction and purification of total phenolics from *C. camphora* roots were systematic optimization. Response surface methodology identified optimal conditions of 71% ethanol, 78 °C, and liquid-to-solid a ratio of 24:1 (mL/g), resulting in a total phenolic content of 3.60 mg/g. Purification with HPD-600 resin followed pseudo-second-order kinetics (*R*^2^ = 0.9987). Twelve phenolic compounds were isolated, seven of which are reported from *C. camphora* roots for the first time. The enriched fraction demonstrated strong antioxidant activities and significant pancreatic lipase inhibitory activity (IC_50_ = 0.80 mg/mL). Although many minor phenolics remain to be characterized, their potential additive or synergistic contributions to the observed bioactivity should not be overlooked. These findings expand the chemical diversity of *C. camphora* roots and suggest their potential as natural antioxidants, while the lipid-lowering activity warrants further validation using cellular and in vivo models.

## Figures and Tables

**Table 1:** Analysis of variance (ANOVA) for the response surface quadratic model.

Source	Sum of Squares	D_f_	Mean Squared	*F*-Value	*p*-Value	Significance
Model	1.46	9	0.1618	150.45	<0.0001	***
*A*	0.0061	1	0.0061	5.62	0.0495	*
*B*	0.0144	1	0.0144	13.43	0.0080	**
*C*	0.0162	1	0.0162	15.06	0.0060	**
*AB*	0.0552	1	0.0552	51.34	0.0002	**
*AC*	0.0342	1	0.0342	31.82	0.0008	**
*BC*	0.0342	1	0.0342	31.82	0.0008	**
A^2^	0.1809	1	0.1809	168.12	<0.0001	***
B^2^	0.4789	1	0.4789	445.19	<0.0001	***
C^2^	0.5077	1	0.5077	471.98	<0.0001	***
Residual	0.0075	7	0.0011			
Lack of Fit	0.0057	3	0.0019	4.01	0.1066	
Pure Error	0.0019	4	0.0005			
Cor Total	1.46	16				
*R* ^2^	0.9949					
Adjusted *R*^2^	0.9882					
CV%	1.02					

* *p* < 0.05; ** *p* < 0.01; *** *p* < 0.001.

**Table 2:** Response surface test factors and levels.

Levels	Extraction Factors		
	A Ethanol Concentration [%]	B Extraction Temperature [°C]	C Liquid-to-Solid Ratio [mL/g]
−1	60	70	15
0	70	80	25
1	80	90	35

## Data Availability

The original contributions presented in this study are included in the article/Supplementary Material. Further inquiries can be directed to the corresponding author(s).

## Supplementary Materials

The following supporting information can be downloaded at: https://www.mdpi.com/article/10.3390/molecules31091550/s1, Table S1. UPLC-Q-TOF-MS/MS identification of phenolic compounds from *C. camphora* roots; Table S2. Box–Behnken design matrix with experimental and predicted values for total phenolic extraction from *C. camphora* roots; Table S3. Kinetic parameters of adsorption models for total phenolics on HPD-600 resin; Figure S1. Diagnostic plots for the response surface quadratic model of total phenolic extraction from *C. camphora* roots. Figure S2. UPLC-ESI-QTOF-MS/MS total ion chromatograms of the phenolic extract from *C. camphora* roots in (A) positive ion mode and (B) negative ion mode; and Spectroscopic Data of Isolated Compounds from *C. camphora* roots.
