# Supplementary material for: Phenolic Compounds from Cinnamomum camphora Roots: Extraction Optimization, Purification, Isolation, and Bioactivity Evaluation
Source: Molecules. 2026 May 6;31(9):1550. doi: 10.3390/molecules31091550 (PMC13164988; doi:10.3390/molecules31091550)
Supplement: Supplementary file 1 [file molecules-31-01550-s001.zip › molecules-4268230-supplementary.pdf]

Supplementary Materials

Phenolic Compounds from Cinnamomum camphora Roots: Extraction Optimization, Purification, Isolation, and Bioactivity Evaluation

Jinhua Long†, Wei Zheng†, Lu Liu, Yuting Pang, Yijia Zhang, Yuping Luan, Nan Xu*

School of Pharmacy, Liaoning University of Traditional Chinese Medicine, Liaoning, China

*Correspondence: Nan Xu (xudanbs@163.com)

†These authors contributed equally to this work and should be considered as co-first authors.

Abstract: The roots of *Cinnamomum camphora* have been traditionally used in folk medicine, yet their phenolic constituents have not been extensively studied. This research aims to optimize the extraction and purification of total phenolics from *C. camphora* roots, isolate their chemical constituents, and evaluate their bioactivities. Response surface methodology optimized the extraction process, resulting in optimal conditions of ethanol concentration of 71%, extraction temperature of 78°C, and a liquid-to-solid ratio of 24 :1 (mL/g), which yielded a total phenolic content of 3.60 mg/g. The extract underwent purification using HPD-600 macroporous resin, and the adsorption kinetics conformed to a pseudo-second-order model (R^2 = 0.9987). Twelve phenolic compounds were isolated, seven of which were identified from *C. camphora* roots for the first time. The enriched fraction exhibited strong antioxidant activities, with DPPH IC_{50} at 107.2 μg/mL, superoxide anion IC_{50} at 141.7 μg/mL, and •OH IC_{50} at 130.7 μg/mL, along with significant pancreatic lipase inhibitory activity (IC_{50} = 0.80 mg/mL). This integrated approach expands the chemical diversity of phenolic constituents in *C. camphora* roots and provides a scientific basis for their potential application as natural antioxidants and lipid-lowering agents.

Keywords: *Cinnamomum camphora*; phenolics; extraction optimization; isolation; pancreatic lipase inhibition

Table S1. UPLC-Q-TOF-MS/MS identification of phenolic compounds from C. camphora roots.

No.	Retention time(min)	Component name	Formula	Mass error (ppm)	Adduct ion	quasi-molecular ion (*m/z*)	MS/MS(*m/z*)
1	2.71	coumarin	$C_9H_6O_2$	-1.36	$[M+H]^+$	147.0446	119.0487,91.0538,65.0381
2	3.37	5-galloylquinic acid	$C_{14}H_{16}O_{10}$	-0.58	$[M-H]^-$	343.0666	191.0561,173.0449,129.0549
3	4.21	syringic acid	$C_9H_{10}O_5$	-2.03	$[M-H]^-$	197.0457	167.0346,149.0232,153.0551,125.0596
4	4.82	5-aminosalicylic acid	$C_7H_7NO_3$	1.32	$[M+H]^+$	152.0515	137.0246,119.0142,93.0344
5	6.07	shikimic acid	$C_7H_{10}O_5$	2.31	$[M-H]^-$	173.0448	155.0356,129.0558,111.0458,85.0651
6	6.93	p-coumaraldehyde	$C_9H_8O_2$	1.34	$[M+H]^+$	149.0603	119.0493,91.0545,65.0386
7	7.32	Gallic acid	$C_7H_6O_5$	1.78	$[M-H]^-$	169.0139	125.0243,97.0298,79.0191,53.0397
8	7.43	4'-*O*-methylcatechin	$C_{16}H_{16}O_5$	1.44	$[M+H]^+$	289.1067	271.0932,245.1107,153.0163
9	7.66	pyrogallol	$C_6H_6O_3$	3.99	$[M-H]^-$	125.0190	97.0297,79.0191,69.0349,51.0240
10	7.91	quercetin 3-*O*-glucoside 7-*O*-rhamnoside	$C_{21}H_{20}O_{11}$	1.53	$[M+H]^+$	449.1088	303.0505,169.0501,139.0404,137.0589
11	8.61	protocatechuic acid 3-*O*-beta-glucoside	$C_{13}H_{16}O_9$	0.95	$[M-H]^-$	315.0725	153.0195,109.0297
12	9.15	catechol	$C_6H_6O_2$	0.96	$[M-H]^-$	109.0241	91.0190，65.0032，41.0031
13	9.82	2,3-dihydroxybenzoic acid	$C_7H_6O_4$	3.26	$[M-H]^-$	153.0139	109.0284,81.0347
14	9.84	taxifolin	$C_{15} H_{12} O_7$	-1.30	$[M+H]^+$	305.0702	153.01830,149.02336,123.04427,231.06519
15	10.25	2,3,6-Trimethoxy-4-methylphenol	$C_{10}H_{14}O_4$	5.02	$[M-H]^-$	197.0764	181.0851,155.01062,127.1124,85.0688
16	10.71	3,4-dihydroxybenzaldehyde	$C_7H_6O_3$	4.61	$[M-H]^-$	137.0317	108.0207,119.0122,92.0257
17	11.17	phenol	C_6H_6O	2.14	$[M-H]^-$	93.0290	65.0395,39.0238
18	12.14	caffeic acid	$C_9H_8O_4$	1.67	$[M-H]^-$	179.0345	135.0453,107.0506,79.0556
19	12.31	3-methoxy-quercetin	$C_{16}H_{12}O_7$	1.71	$[M+H]^+$	317.0659	302.0422,274.0477,153.0186,139.0398,121.0286
20	12.47	myricetin	$C_{15}H_{10}O_8$	1.58	$[M-H]^-$	317.0294	273.0263,255.0259,227.0333,151.0039,
21	12.86	4',5,7-trihydroxy-3'-methoxyflavone	$C_{16}H_{12}O_6$	1.07	$[M+H]^+$	301.0674	286.0465,229.0464,203.0308,153.0166
22	15.79	7-methoxy-2H-chromen-2-one	$C_{10}H_8O_3$	-2.82	$[M+H]^+$	177.0603	147.0439,119.0486,91.0537
23	16.47	vanillic acid	$C_8H_8O_4$	3.59	$[M-H]^-$	167.0345	123.0453,152.0117,108.0219

TableS1. Cont.

24	18.45	ethyl 4'-hydroxy-3'-methoxycinnamate	$C_{12}H_{14}O4$	-0.91	$[M+H]^+$	221.1022	177.0919,149.0969,123.0813,77.0394
25	19.00	quercetin	$C_{15}H_{10}O_7$	-0.91	$[M-H]^-$	301.0294	273.0402,255.0295,245.0453,227.0348,179.0352,151.0035,135.0450,121.0294,107.0136
26	19.30	eriodictyol	$C_{15}H_{12}O_6$	2.83	$[M-H]^-$	287.0501	259.0615,243.0666,269.0469,201.0561,125.0248,109.0298
27	19.70	salicylaldehyde	$C_7H_6O_2$	-1.65	$[M-H]^-$	121.0241	91.0187,73.0086,65.0031
28	19.93	Dimethyltetrahydrocurcumin	$C_{23}H_{28}O_6$	0.49	$[M+H]^+$	401.1957	385.1644,372.1929,151.0751
29	20.75	kaempferol	$C_{15}H_{10}O_6$	-1.86	$[M-H]^-$	285.0345	241.0500,217.0499,199.0399,151.0034,133.0290,107.0136
30	21.90	luteolin	$C_{15}H_{10}O_6$	1.74	$[M-H]^-$	285.0345	151.0039,125.0246,121.0292
31	21.92	kobusin	$C_{21}H_{22}O_6$	-1.96	$[M+H]^+$	371.1491	339.1226,321.1119,293.1168,261.0908,137.0595
32	22.59	ferulic acid	$C_{10}H_{10}O_4$	-1.57	$[M-H]^-$	193.0501	149.0604,105.0703,119.0500,105.0703
33	24.83	DihydrocurcuMin	$C_{21}H_{22}O_6$	-0.96	$[M+H]^+$	371.1491	339.1226,321.1085,293.1108,261.0887,261.0887,217.0855
34	25.14	curcumin	$C_{21}H_{20}O_6$	-0.54	$[M+H]^+$	369.1378	339.1226,247.0963,217.0856,151.0751
35	25.26	(2*E*)-3-(4-Hydroxy-3,5-dimethoxyphenyl)acrylaldehyde	$C_{11}H_{12}O_4$	-1.43	$[M+H]^+$	209.0805	177.0543,149.0595,121.0646,77.03878
36	25.56	4-*O*-Acetylcaffeic acid	$C_{11}H_{10}O_5$	-2.26	$[M-H]^-$	221.0396	177.0555,147.0449,117.0341
37	25.77	kaempferol	$C_{15}H_{12}O_6$	-0.90	$[M-H]^-$	287.0634	273.0402,255.0295,245.0453,227.0348,179.0352,151.0035,135.0450,121.0294,107.0136
38	27.20	5,7-Dihydroxyflavanone	$C_{15}H_{12}O_4$	-0.75	$[M-H]^-$	255.0654	211.0763,185.0605,177.0190,151.0033,107.0139,103.0550,65.0395
39	27.23	6-Tridecylsalicylic acid	$C_{20}H_{32}O_3$	-0.93	$[M+H]^+$	321.2422	303.2317,285.2211,267.2103,185.1322,121.1010,95.0852
40	28.07	7-Hydroxy-4′-methoxyisoflavone	$C_{16}H_{12}O_4$	2.60	$[M+H]^+$	269.0864	254.0579,226.0628,103.0545,77.0389
41	28.25	apigenin	$C_{15}H_{10}O_5$	-1.48	$[M-H]^-$	269.0396	225.0554,201.0554,181.0655,151.0022,117.0343,93.0345
42	28.55	naringenin	$C_{15}H_{12}O_5$	-1.11	$[M-H]^-$	271.0602	227.0711,201.0555,177.0190,151.0033,119.0489,107.0136,93.0343
43	28.71	esculin	$C_{15}H_{16}O_9$	1.10	$[M+H]^+$	341.1023	179.03391,133.02850,123.04425
44	28.94	scopoletin	$C_{10}H_8O_4$	-0.96	$[M+H]^+$	193.0552	178.0264,165.0551,133.0288
45	29.37	5-*O*-Methylflavone	$C_{16}H_{12}O_3$	1.68	$[M+H]^+$	253.2702	224.0679,196.0650,151.0728,121.0684

Table S2. Box–Behnken design matrix with experimental and predicted values for total phenolic extraction from *C. camphora* roots.

Run	Extraction Factors			TPC [mg/g]
	A<br>ethanol concentration [%]	B<br>extraction temperature [°C]	C<br>liquid-to-solid ratio [mL/g]	
1	-1	1	0	2.91
2	0	-1	-1	3.15
3	0	0	0	3.60
4	0	0	0	3.64
5	0	1	-1	2.86
6	1	0	-1	3.22
7	1	1	0	3.18
8	0	0	0	3.61
9	0	0	0	3.61
10	-1	-1	0	3.21
11	0	1	1	2.91
12	0	0	0	3.65
13	-1	0	1	3.10
14	0	-1	1	2.83
15	1	0	1	2.99
16	1	-1	0	3.01
17	-1	0	-1	2.96

Table S3. Kinetic parameters of adsorption models for total phenolics on HPD-600 resin.

Model	Parameter	Value
Pseudo-first-order	k_1 (min^{-1})	0.0234
	R^2	0.9853
Pseudo-second-order	k_2 [g/(mg·min)]	0.000291
	Qe, calc (mg/g)	34.6
	R^2	0.9987
Intraparticle diffusion (Stage 1)	k_{i1} [mg/(g·min$^{1/2}$)]	3.505
	C_1	-8.15
	$R^2{}_1$	0.9613
Intraparticle diffusion (Stage 2)	k_{i2} [mg/(g·min$^{1/2}$)]	0.733
	C_2	23.91
	$R^2{}_2$	0.9921

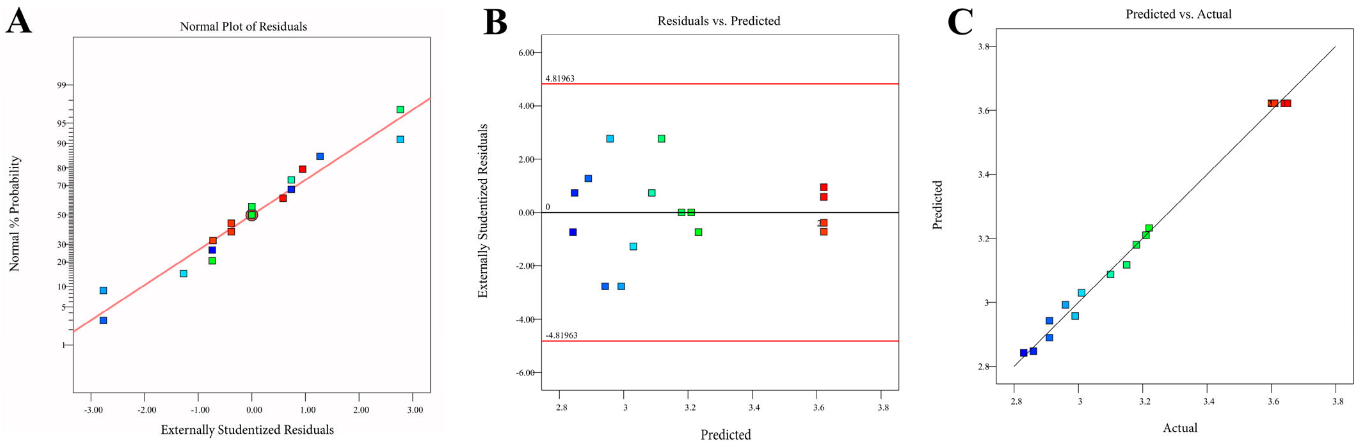

Figure S1. Diagnostic plots for the response surface quadratic model of total phenolic extraction from *C. camphora* roots. (A) Normal probability plot of residuals: points approximately follow a straight line, confirming the assumption of normality. (B) Residuals vs. predicted values: points are randomly scattered around zero without a clear trend, indicating constant variance (homoscedasticity). (C) Predicted vs. actual values: points cluster tightly around the 45° diagonal line (R^2 = 0.9949), demonstrating good predictive capability of the model.

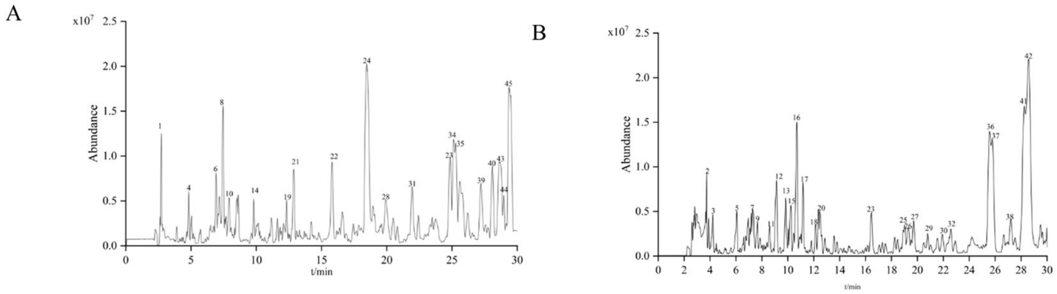

Figure S2. UPLC-ESI-QTOF-MS/MS total ion chromatograms (TIC) of the phenolic extract from C. camphora roots. (A) Positive ion mode; (B) negative ion mode. Chromatographic conditions: Agilent Poroshell 120 EC-C18 column (4.6 mm × 100 mm, 2.7 μm); mobile phase: acetonitrile (A) and 0.1% formic acid in water (B); gradient elution: 0–10 min, 5–50% A; 10–25 min, 50–70% A; 25–30 min, 70–100% A; flow rate: 0.4 mL/min; injection volume: 5 μL. Mass spectrometry: dual AJS ESI source; mass range m/z 50–1000. The 45 tentatively identified compounds (Table S1) are labeled with their peak numbers.

Spectroscopic Data of Isolated Compounds from C. camphora Roots

Isolation of Chemical Constituents

The enriched phenolic fraction (26.5 g) was subjected to silica gel column chromatography (CC) and eluted with a gradient of petroleum ether–ethyl acetate (PE–EtOAc, 100:0 → 0:100, v/v) to yield eight fractions (Fr. A–H).

Compound 1 (12.5 mg) was crystallized from Fr. B (3.1 g).

Fr. C (2.8 g) was fractionated by preparative HPLC (MeOH–H_2O = 85:15, 1.5 mL/min) to yield **compounds 2** (9.8 mg) and 3 (5.7 mg).

Fr. D (3.2 g) was subjected to repeated silica gel CC (PE–EtOAc, 80:20 → 0:100) and Sephadex LH-20 CC to afford **compounds 4** (6.7 mg), **5** (7.5 mg), and **6** (5.5 mg).

Compounds 7 (5.4 mg) and **8** (5.1 mg) were isolated from Fr. F (3.8 g) by preparative HPLC (MeOH–H_2O = 80:20, 1.5 mL/min).

Compounds 9 (11.2 mg) and **10** (7.4 mg) were obtained from Fr. G (5.4 g) by Sephadex LH-20 CC and preparative HPLC (MeOH–H_2O = 77:23, 1.5 mL/min).

Fr. H (5.6 g) was fractionated by Sephadex LH-20 CC to yield four subfractions (Fr. H1–H4). **Compounds 11** (5.1 mg) and **12** (6.4 mg) were purified from Fr. H2 by preparative HPLC (MeOH–H_2O = 75:25, 1.5 mL/min).

All isolated compounds were identified by HR-ESI-MS and 1D NMR analyses.

identification of Chemical Constituents

Compound 1 (Ferulic acid): HR-ESI-MS *m/z* 371.1491 $[M-H]^-$. ^{1}H-NMR (400 MHz, Methanol-d_4) δ: 7.59 (1H, d, J = 15.9 Hz, H-7), 7.17 (1H, br s, H-2), 7.05 (1H, br d, J = 8.0 Hz, H-6), 6.80 (1H, d, J = 8.0 Hz, H-5), 6.30 (1H, d, J = 15.9 Hz, H-8), 3.91 (3H, s, 3-OCH_3). ^{13}C-NMR (100 MHz, Methanol-d_4) δ: 115.0 (C-1), 126.4 (C-2), 149.1 (C-3), 148.0 (C-4), 122.6 (C-5), 110.3 (C-6), 145.5 (C-7), 114.5 (C-8), 169.6 (C-9), 55.0 (3-OCH_3).

Compound 2 (Caffeic acid): HR-ESI-MS *m/z* 179.0345 $[M-H]^-$. ^{1}H-NMR (400 MHz, Methanol-d_4) δ: 7.53 (1H, d, J = 15.9 Hz, H-7), 7.04 (1H, d, J = 2.1 Hz, H-2), 6.93 (1H, dd, J = 8.2, 2.1 Hz, H-6), 6.78 (1H, d, J = 8.2 Hz, H-5), 6.22 (1H, d, J = 15.9 Hz, H-8). ^{13}C-NMR (100 MHz, Methanol-d_4) δ: 123.0 (C-1), 128.0 (C-2), 146.9 (C-3), 149.5 (C-4), 115.2 (C-5), 115.9 (C-6), 147.1 (C-7), 116.3 (C-8), 171.3 (C-9).

Compound 3 (Scopoletin): HR-ESI-MS *m/z* 193.0552 $[M+H]^+$. ^{1}H-NMR (400 MHz, DMSO-d_6) δ: 3.91 (3H, s, -OCH_3), 6.18 (1H, d, J = 9.5 Hz, H-3), 6.80 (1H, s, H-8), 7.21 (1H, s, H-5), 7.85 (1H, d, J = 9.5 Hz, H-4), 8.76 (1H, s, 7-OH). ^{13}C-NMR (150 MHz, DMSO-d_6) δ: 55.8 (-OCH_3), 102.8 (C-8), 109.0 (C-3), 111.2 (C-5), 112.4 (C-4a), 143.7 (C-4), 145.0 (C-7), 150.2 (C-8a), 150.9 (C-6), 160.4 (C-2).

Compound 4 (Vanillic acid): HR-ESI-MS *m/z* 167.0345 $[M-H]^-$. ^{1}H-NMR (400 MHz, Methanol-d_4) δ: 7.54 (1H, d, J = 1.8 Hz, H-1), 6.81 (1H, d, J = 8.4 Hz, H-5), 7.53 (1H, dd, J = 8.4, 1.8 Hz, H-6), 3.88 (3H, s, -OCH_3). ^{13}C-NMR (100 MHz, Methanol-d_4) δ: 122.1 (C-1), 114.4 (C-2), 147.2 (C-3), 151.1 (C-4), 112.4 (C-5), 123.8 (C-6), 168.9 (C-7), 55.0 (-OCH_3).

Compound 5 (Gallic acid): HR-ESI-MS *m/z* 169.0139 $[M-H]^-$. ^{1}H-NMR (400 MHz, Methanol-d_4) δ: 7.06 (2H, s, H-2, H-6). ^{13}C-NMR (100 MHz, Methanol-d_4) δ: 122.0 (C-1), 110.3 (C-2, C-6), 146.4 (C-3, C-5), 139.6 (C-4), 170.4 (-COOH).

Compound 6 (Catechol): HR-ESI-MS *m/z* 109.0241 $[M-H]^-$. ^{1}H-NMR (400 MHz, Methanol-d_4) δ: 6.64 (2H, m, H-2,6), 6.55 (2H, m, H-3,5). ^{13}C-NMR (100 MHz, Methanol-d_4) δ: 119.5 (C-2,6), 115.0 (C-3,5) ,144.9 (C-4).

Compound 7 (Kaempferol): HR-ESI-MS *m/z* 285.0345 $[M-H]^-$. ^{1}H-NMR (400 MHz, Methanol-d_4) δ: 12.50 (1H, s, 5-OH), 10.81 (1H, s, 7-OH), 10.13 (1H, s, 4′-OH), 9.43 (1H, s, 3′-OH), 8.06 (2H, d, J = 7.9, 2.1 Hz, H-2′, H-6′), 6.94 (2H, d, J = 7.9, 2.1 Hz, H-3′, H-5′), 6.45 (1H, d, J = 2.1 Hz, H-6), 6.17 (1H, d, J = 2.1 Hz, H-8). ^{13}C-NMR (100 MHz, Methanol-d_4) δ: 146.9 (C-2), 135.8 (C-3), 176.0 (C-4), 156.3 (C-5), 98.3 (C-6), 164.0 (C-7), 93.6 (C-8), 160.8 (C-9), 103.1 (C-10), 121.8 (C-1′), 129.6 (C-2′, C-6′), 115.5 (C-3′, C-5′), 159.3

(C-4′).

Compound 8 (Naringenin): HR-ESI-MS *m/z* 271.0602 $[M-H]^-$. ^{1}H-NMR (400 MHz, Methanol-d_4) δ: 7.35 (1H, s, H-11), 6.83 (1H, s, H-3), 3.91 (3H, s, 9-OCH_3), 3.85 (1H, s, 10-OH), 3.31 (2H, t, J = 6.8 Hz, 2-CH_2), 2.89 (2H, t, J = 6.8 Hz, 1-CH_2). ^{13}C-NMR (100 MHz, Methanol-d_4) δ: 78.4 (C-2), 41.9 (C-3), 196.3 (C-4), 163.4 (C-5), 94.9 (C-6), 166.6 (C-7), 95.8 (C-8), 162.8 (C-9), 101.7 (C-10), 128.8 (C-1′), 128.3 (C-2′,6′), 115.1 (C-3′,5′), 157.7 (C-4′).

Compound 9 (Dihydrokaempferol): HR-ESI-MS *m/z* 287.0634 $[M-H]^-$. ^{1}H-NMR (400 MHz, Methanol-d_4) δ: 7.35 (2H, d, J = 8.4 Hz, H-2′,6′), 6.83 (2H, d, J = 8.4 Hz, H-3′,5′), 5.93 (1H, d, J = 2.0 Hz, H-8), 5.88 (1H, d, J = 2.0 Hz, H-6), 4.98 (1H, d, J = 12.0 Hz, H-3), 4.54 (1H, d, J = 11.6 Hz, H-2). ^{13}C-NMR (100 MHz, Methanol-d_2) δ: 85.0 (C-2), 73.6 (C-3), 198.5 (C-4), 165.3 (C-5), 96.3 (C-6), 168.7 (C-7), 97.3 (C-8), 164.5 (C-9), 101.8 (C-10), 129.3 (C-1′), 130.4 (C-2′,6′), 116.1 (C-3′,5′), 159.2 (C-4′).

Compound 10 (Taxifolin): HR-ESI-MS *m/z* 305.0702 $[M+H]^+$. ^{1}H-NMR (400 MHz, Methanol-d_4) δ: 6.97 (1H, d, J = 2.0 Hz, H-2′), 6.86–6.79 (3H, m, H-5′, H-6′, H-8), 5.88 (1H, s, H-6), 4.91 (1H, d, J = 11.2 Hz, H-2), 4.50 (1H, d, J = 11.2 Hz, H-3). ^{13}C-NMR (100 MHz, Methanol-d_4) δ: 198.3 (C-4), 168.8 (C-5), 165.3 (C-7), 164.5 (C-3′/C-4′), 147.1 (C-3′), 146.3 (C-4′), 129.9 (C-1′), 120.9 (C-6′), 116.1 (C-2′), 115.9 (C-5′), 101.8 (C-10), 97.3 (C-6), 96.3 (C-8), 85.1 (C-2), 73.7 (C-3).

Compound 11 (Luteolin): HR-ESI-MS *m/z* 285 $[M-H]^-$. ^{1}H-NMR (400 MHz, Methanol-d_4) δ: 7.37 (2H, d, J = 7.7 Hz, H-2′,6′), 6.88 (1H, d, J = 8.1 Hz, H-5′), 6.51 (1H, s, H-3), 6.42 (1H, s, H-8), 6.17 (1H, s, H-6). ^{13}C-NMR (100 MHz, Methanol-d_4) δ: 163.8 (C-2), 101.6 (C-3), 181.6(C-4), 160.1 (C-5), 97.8 (C-6), 164.1 (C-7),92.7 (C-8), 157.2 (C-9), 103.1 (C-10), 121.4 (C-1′), 111.9 (C-2′), 144.8 (C-3′), 148.7 (C-4′), 114.5 (C-5′), 118.0 (C-6′) .

Compound 12 (Quercetin): HR-ESI-MS *m/z* 301.0294 $[M-H]^-$. ^{1}H-NMR (400 MHz, Methanol-d_4) δ: 6.18 (1H, d, J = 2.0 Hz, H-6), 6.40 (1H, d, J = 2.0 Hz, H-8), 7.67 (1H, d, J = 2.2 Hz, H-2′), 6.88 (1H, d, J = 8.5 Hz, H-5′), 7.54 (1H, dd, J = 2.2, 8.5 Hz, H-6′), 9.57 (1H, br s, 3′-OH), 12.48 (1H, s, 5-OH), 10.76 (1H, br s, 7-OH), 9.34 (1H, br s, 4′-OH). ^{13}C-NMR (100 MHz, Methanol-d_4) δ: 147.9 (C-2), 135.9 (C-3), 176.0 (C-4), 160.9 (C-5), 98.4 (C-6), 164.1 (C-7), 93.6 (C-8), 156.3 (C-9), 103.2 (C-10), 122.2 (C-1′), 115.8 (C-2′), 145.3 (C-3′), 147.0 (C-4′), 115.3 (C-5′), 120.2 (C-6′).
